# Acute NaCl Loading Reveals a Higher Blood Pressure for a Given Serum Sodium Level in African American Compared to Caucasian Adults

**DOI:** 10.3389/fphys.2018.01354

**Published:** 2018-10-01

**Authors:** Megan M. Wenner, Erin P. Paul, Austin T. Robinson, William C. Rose, William B. Farquhar

**Affiliations:** Department of Kinesiology and Applied Physiology, University of Delaware, Newark, DE, United States

**Keywords:** osmolality, RAAS, blood pressure, race, hypertonic saline

## Abstract

**Purpose:** African American individuals are more prone to salt-sensitive hypertension than Caucasian individuals. Small changes in serum sodium (Na^+^) result in increased blood pressure (BP). However, it remains unclear if there are racial differences in BP responsiveness to increases in serum Na^+^. Therefore, the purpose of this investigation was to determine if African American adults have altered BP responsiveness to acute changes in serum Na^+^ compared to Caucasian adults.

**Methods:** We measured beat-by-beat BP, serum Na^+^, plasma renin activity (PRA), angiotensin II (Ang II), and aldosterone (Aldo) during a 60-min 3% NaCl infusion (hypertonic saline infusion, HSI) in 39 participants (19 African Americans, age: 23 ± 1, 20 Caucasians, age: 25 ± 1). Data reported as African American vs. Caucasian cohort, mean ± SEM.

**Results:** Baseline BP and serum Na^+^ were similar between groups and increased during HSI in both African American and Caucasian participants (*p* < 0.01). However, the peak change in serum Na^+^ was greater in African American participants (Δ5.8 ± 0.34 vs. Δ4.85 ± 0.38 mmol/L, *p* = 0.03). There was a significant group effect (*p* = 0.02) and an interaction between race and serum Na^+^ on systolic BP (*p* = 0.02). Larger categorical changes in serum Na^+^ corresponded to changes in systolic BP (*p* < 0.01) and African American participants demonstrated greater systolic BP responses for a given categorical serum Na^+^ increase (*p* < 0.01). Baseline Aldo was lower in African American adults (7.2 ± 0.6 vs. 12.0 ± 1.9 ng/dL, *p* = 0.03), there was a trend for lower baseline PRA (0.59 ± 0.9 vs. 1.28 ± 0.34 ng/mL/h, *p* = 0.07), and baseline Ang II was not different (14.2 ± 1.8 vs. 18.5 ± 1.4 pg/mL, *p* = 0.17). PRA and Aldo decreased during the HSI (*p* ≤ 0.01), with a greater decline in PRA (Δ–0.31 ± 0.07 vs. Δ–0.85 ± 0.25 ng/mL/h, *p* < 0.01) and Aldo (Δ–2.5 ± 0.5 vs. Δ–5.0 ± 1.1 ng/dL, *p* < 0.01) in Caucasian participants. However, the racial difference in PRA (*p* = 0.57) and Aldo (*p* = 0.59) reduction were no longer significant following baseline covariate analysis. **Conclusion:** African American individuals demonstrate augmented serum Na^+^ to an acute hypertonic saline load and greater systolic BP responsiveness to a given serum Na^+^. The altered BP response may be attributable to lower basal PRA and Aldo and a subsequently blunted RAAS response during the HSI.

## Introduction

In the United States, African American individuals have a higher prevalence and severity of hypertension, and develop hypertension at an earlier age than Caucasian individuals (Williams et al., 2014; Fryar et al., 2017). Furthermore, African American adults have greater rates of premature hypertensive complications such as chronic kidney disease, stroke, and coronary heart disease (Williams et al., 2014; Fryar et al., 2017). The relation between dietary salt and BP is well established (Appel et al., 2011; He et al., 2013) and African American individuals have been shown to be more prone to the adverse cardiovascular effects of chronic high dietary salt although the mechanisms for these disparities remain to be fully elucidated (Williams et al., 2014; Elijovich et al., 2016).

Increased dietary salt intake can elicit small, but consequential changes in plasma sodium (Na^+^) concentration (He et al., 2005; He and Macgregor, 2012) and small changes in plasma Na^+^ are associated with elevated BP and vascular dysfunction (Dickinson et al., 2011, 2014; Suckling et al., 2012; Matthews et al., 2015). The RAAS is comprised of fluid regulatory hormones that directly control Na^+^ excretion by the kidneys. Increased RAAS activation contributes to greater Na^+^ reabsorption (Hall et al., 1990). African American individuals have lower basal PRA and Aldo levels (Levy et al., 1977; Grim et al., 1979; Luft et al., 1979; Sever et al., 1979; He et al., 1998, 1999; Rifkin et al., 2014). Therefore, lower baseline levels of RAAS hormones may decrease African American individuals’ ability to buffer acute Na^+^ challenges and regulate BP. This is important because altered BP and RAAS hormone responses may partially explain why African American adults are at a greater risk for experiencing adverse cardiovascular effects with high dietary salt. However, previous studies examining the role of RAAS on the racial differences to salt have primarily been done in hypertensive adults (He and MacGregor, 2004) or have not accounted for baseline differences in RAAS when comparing responses to salt manipulation (Luft et al., 1991).

We have previously used HSI (3% NaCl) to acutely increase serum Na^+^ and have found increased sympathetic nerve activity and altered baroreflex regulation of BP control (Farquhar et al., 2005, 2006; Wenner et al., 2007; Greaney et al., 2010). These findings do not occur with isovolumic isotonic saline infusion, suggesting the relation between serum Na^+^ concentration and BP is independent of sodium-induced expansion of the extracellular fluid volume (Farquhar et al., 2005, 2006). However, it remains unknown if there is racial difference in BP responsiveness to a given change in serum Na^+^ level. Accordingly, the purpose of the present study was to determine whether serum Na^+^, BP, and RAAS responses to an acute Na^+^ and volume load via HSI differ between young, normotensive African American and Caucasian adults. We hypothesized that African American individuals would have greater BP responsiveness for a given change in serum Na^+^. We also hypothesized lower basal levels of RAAS in African American individuals would contribute to a lesser reduction in the RAAS in response to the HSI (i.e., similar directional response, but reduced magnitude).

## Materials and Methods

### Ethical Approval

The Human Subjects Institutional Review Board of the University of Delaware approved this study (HS#05-147). Participants gave written consent prior to participation. This study conformed to the standards set by the Declaration of Helsinki; however, this physiology study was not registered.

### Participants

Thirty-nine participants with normal resting BP were recruited–19 African Americans (9 males and 10 females) and 20 Caucasians (15 males and 5 females)–for the study. Only participants that self-reported both parents as Caucasian or both parents as African American were included in the study.

### Screening Visit

All participants filled out a Physical Activity Readiness Questionnaire and medical history form (Franklin, 2000). A fasting blood sample was taken to measure liver enzymes (alanine aminotranferase; ALT and aspartate aminotransferase; AST), kidney function (creatinine and blood urea nitrogen; BUN), a lipid profile, fasting glucose, and electrolytes, and a complete blood count. Height and weight were measured and body mass index (BMI) was calculated. Body fat percent was estimated using the skin fold technique (Franklin, 2000). Resting BP and ECG were measured (GE Dash 2000 GE Medical Systems, Milwaukee, WI, United States).

### Experimental Visit

To ensure adequate hydration, participants were instructed to drink 600 ml of water during the morning, mid-day, and night the day before the experimental visit, and another 600 ml of water the morning of the experimental visit. Participants were instructed to avoid table salt and foods that are high in sodium for 24 h preceding the experimental visit, and were provided a list of common foods that are high in Na^+^ to avoid (Takamata et al., 1995; Stachenfeld et al., 1996, 1998, 2001; Calzone et al., 2001; Stachenfeld and Keefe, 2002). They were also instructed to avoid caffeine, exercise, and alcohol for at least 12 h before the visit. Females were tested during the early follicular phase of the menstrual cycle (self-reported). Upon arrival, participants emptied their bladder and a urine sample was collected to measure specific gravity (TS Meter Hand-held Refractometer, Reichert Inc., Depew, NY, United States) to confirm adequate hydration, and to rule out pregnancy in female participants (hCG rapid one-step test, Mainline Technology, Ann Arbor, Michigan). Participants were then equipped for measurement of single lead ECG, and oscillometric brachial BP. BP was taken in triplicate. A finger cuff was placed for analysis of beat-by-beat BP via servo-controlled photoplethysmography (Finometer, Finapres Medical Systems, Netherlands). An intravenous catheter was placed in the left arm for the HSI and the right arm for blood draws. The HSI is a large Na^+^ and volume load, which has been shown to cause an increase in plasma Na^+^ concentration and osmolality over a short period of time (Farquhar et al., 2005, 2006; Wenner et al., 2007; Greaney et al., 2010).

### Experimental Protocol

At baseline, Finometer derived BP and HR were measured during 5 min of paced breathing at 0.25 Hz, and a blood sample was obtained. After baseline, a 60-min HSI began at a rate of 0.15 ml/kg/min. BP and HR were measured during 5 min of paced breathing, every 20 min into the HSI. Blood was drawn and analyzed for electrolytes, osmolality and hematocrit every 15 min and for PRA, Ang II, Aldo, and NE every 30 min.

### Blood Analysis

Blood was drawn and placed in: (1) aprotinin-treated 4°C EDTA-sodium metabisulfite vacutainers for analysis of plasma NE; (2) K3 EDTA containing vacutainers for the analysis of PRA, plasma Ang II, Aldo, osmolality and hematocrit; and (3) no-additive vacutainers for the analysis of serum Na^+^. Whole blood samples were spun at 450 × *g* for 15 min at 4°C for separation of serum and plasma (Allegra X-22R, Beckman Coulter). Whole blood was placed into capillary tubes (Clay Adams Brand, Becton Dickinson, Parsippany, NJ, United States) and spun in a microcentrifuge to measure hematocrit. Plasma osmolality (model 3D3 Osmometer, Advanced Instruments, Norwood, MA, United States) and serum electrolytes (EasyElectrolytes Analyzer, Medica, Bedford, MA, United States) were measured immediately. The remaining plasma samples were frozen at -80°C for future analysis of NE, PRA, Ang II, and Aldo. NE was analyzed by HPLC at the Mayo Clinic Mayo Medical Laboratories and the RAAS hormones were analyzed by radioimmunoassay at the Wake Forest University Biomarker Analytical Core. The study design involved placing two intravenous catheters. We did not successfully place bilateral catheters in all participants. In cases in which only one catheter was placed, we used this I.V. catheter for HSI. Other difficulties included inadequate sample volume and cracked tubes. Therefore, not all blood samples were analyzed. The total number of participants reported for hormone data are listed in each figure legend. All samples were analyzed in triplicate. Statistics were run on plasma volume-corrected hormone concentrations (further detail below).

### Data Analysis

ECG and BP were recorded by WinDaq recording software (DATAQ Instruments, Akron, OH, United States). The respective signal peaks were detected for determination of HR and BP. Upper-arm BP was taken to confirm Finometer-derived BP at 20, 40, and 60 min during the HSI. Hormones were corrected for the infusion-induced increase in plasma volume by multiplying the hormone values by the percent change in plasma volume [calculated from hematocrit (Van Beaumont, 1972)] at 30 and 60 min. Based on our previous HSI studies (Farquhar et al., 2005, 2006; Wenner et al., 2007; Greaney et al., 2010), we categorized changes in serum Na^+^ of <3 mmol/L as *moderate*, 3–5 mmol/L as *intermediate*, and >5 mmol/L as *large*.

### Statistical Analysis

All data were reported as mean ± SEM. Independent sample *t*-tests were used to analyze baseline racial differences in participants, and differences in peak responses [change scores (Δ) relative to baseline values]. Alternatively, when baseline differences in measures existed, the respective baseline value was used as a covariate and analyzed using ANCOVA. Two-way ANOVA was used to examine main effects of time and race for responses during the HSI. *Post hoc* analysis was completed using Tukey’s HSD when appropriate (significant omnibus *F*). A mixed model analysis was used to assess the relation between race and serum Na^+^ systolic BP. SBP was set as the dependent variable, race was used as the fixed effect, and serum Na^+^ as a covariate. Categorical data were analyzed by the Pearson’s chi-square test (goodness of fit). For all parametric statistical tests, the data was screened for conformity to required statistical assumptions. The α was set at 0.05 for all outcome measures. Statistical analyses were completed using IBM SPSS (24.0).

## Results

Participant characteristics are presented in **Table 1**. African American participants had a lower BUN compared to Caucasian participants. However, the values were within clinically acceptable limits.

**Table 1:** Participant characteristics. Racial differences compared using independent *t*-tests.

Variable	African American	Caucasian	
	*n* = 19	*n* = 20	*p*-value
Age (years)	23 ± 1	25 ± 1	0.289
Sex (F/M)	(10/9)	(5/15)	0.071
SBP (mmHg)	117 ± 2	120 ± 3	0.454
DBP (mmHg)	73 ± 2	74 ± 2	0.514
BMI (kg/m^2^)	25.7 ± 0.8	24.0 ± 0.7	0.264
Body fat (%)	16.8 ± 1.7	15.5 ± 1.4	0.559
Cholesterol (mg/dL)	182 ± 7	180 ± 8	0.898
HDL (mg/dL)	62 ± 4	60 ± 3	0.815
LDL (mg/dL)	105 ± 6	102± 7	0.687
Triglycerides (mg/dL)	74 ± 9	92 ± 8	0.145
Glucose (mg/dL)	86 ± 2	86 ± 2	0.754
Creatinine (mg/dL)	0.98 ± 0.05	1.05 ± 0.04	0.368
BUN (mg/dL)	11.8 ± 0.5	15.3 ± 1.0	0.004
ALT (unit/L)	29.2 ± 2.9	32.9 ± 3.5	0.425
AST (unit/L)	27.7 ± 2.3	31.1 ± 2.2	0.292

The proportion of females in the African American cohort was 53% compared to 25% in the Caucasian cohort. Sex distribution was compared using non-parametric testing and was not statistically different (*p* = 0.07). Participants were well matched for all other variables. There were not differences in urine specific gravity between the groups suggesting hydration status was not a confounding factor (1.020 ± 0.002 in African American individuals vs. 1.015 ± 0.002 in Caucasian individuals, *p* = 0.09). Estimated glomerular filtration rate was calculated from serum creatinine using the CKD-EPI equation and was normal (>90 mL/min/1.73 m^2^) in both groups (Stevens and Levey, 2009).

The HSI provided a robust stimulus, as demonstrated by increases in serum Na^+^, plasma osmolality, systolic and diastolic BP, and heart rate in both groups, while hematocrit decreased (**Table 2**, all *p* < 0.001). Of note, the peak Na^+^ change in African American participants was greater compared to Caucasian participants (Δ5.8 ± 0.3 vs. Δ4.8 ± 0.3; *p* = 0.03). There was also a significant interaction effect for systolic BP (*p* = 0.05). The peak systolic BP was Δ23.2 ± 2.6 in African American participants compared to Δ16.4 ± 2.5 in Caucasian participants (*p* = 0.07).

**Table 2:** Cardiovascular responses to hypertonic saline infusion. Statistical comparisons made using two-way ANOVA.

Variable	Race	Baseline	HSI time-pt. 1	HSI time-pt. 2	HSI time-pt. 3	Statistics
Serum Na^+^ (mmol/L)	African American	134.0 ± 0.3	136.9 ± 0.4	139.5 ± 0.4	139.8 ± 0.4	time (*p* < 0.001)
	Caucasian	135.1 ± 0.6	137.3 ± 0.4	139.8 ± 0.4	139.9 ± 0.3	race (*p* = 0.387)
						interaction (*p* = 0.293)
Plasma Osm (mOsm/kg)	African American	285.8 ± 0.8	290.4 ± 0.8	294.2 ± 0.9	295.5 ± 0.9	time (*p* < 0.001)
	Caucasian	288.4 ± 0.6	291.8 ± 0.6	296.1 ± 0.6	297.3 ± 0.5	race (*p* = 0.069)
						interaction (*p* = 0.293)
Hematocrit (%)	African American	39.7 ± 1.0	37.7 ± 1.0	36.0 ± 1.0	36.1 ± 1.0	time (*p* < 0.001)
	Caucasian	40.6 ± 0.8	38.1 ± 0.7	36.8 ± 0.7	36.3 ± 0.7	race (*p* = 0.546)
						interaction (*p* = 0.248)
Systolic BP (mmHg)	African American	124.0 ± 3.1	136.1 ± 3.2	143.9 ± 3.4	147.2 ± 3.9	time (*p* < 0.001)
	Caucasian	124.8 ± 3.3	133.0 ± 4.0	139.5 ± 4.3	141.2 ± 4.9	race (*p* = 0.542)
						interaction (*p* = 0.054)
Diastolic BP (mmHg)	African American	63.4 ± 2.4	64.3 ± 2.5	68.3 ± 2.4	70.5 ± 2.6	time (p < 0.001)
	Caucasian	63.6 ± 2.1	63.4 ± 2.2	66.9 ± 2.1	68.0 ± 2.2	race (*p* = 0.708)
						interaction (*p* = 0.424
Heart rate (bpm)	African American	60.4 ± 1.8	61.8 ± 1.9	62.0 ± 1.9	64.3 ± 1.9	time (*p* < 0.001)
	Caucasian	60.1 ± 1.6	60.7 ± 1.6	61.5 ± 1.6	63.2 ± 1.8	race (*p* = 0.752)
						interaction (*p* = 0.863)

**FIGURE 1:**
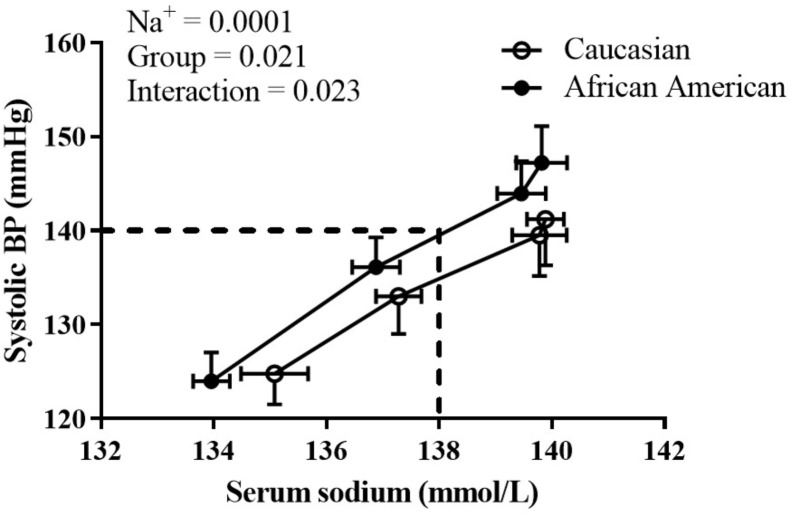
The influence of race and serum Na^+^ on systolic BP. At a given serum Na^+^, African American adults had a greater systolic BP. Statistically significant main effects and interaction listed on graph; all *p* < 0.05. Data presented as mean ± SEM.

**FIGURE 2:**
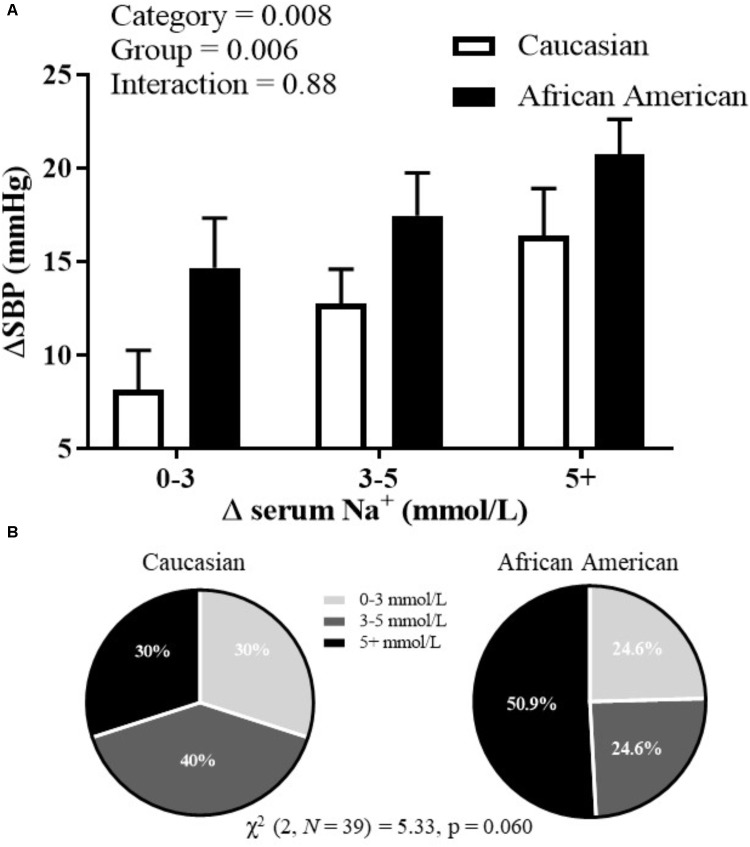
**(A)** Systolic BP changes corresponding to different categorical levels of changes in serum sodium; and **(B)** proportion of categorical serum sodium changes for African American and Caucasian participants. Statistically significant main or interaction effect where *p* < 0.05. Data presented as mean ± SEM.

During the HSI, BP responses were significantly influenced by both race (*p* = 0.02) and serum Na^+^ (*p* < 0.001) (**Figure 1**; mixed model analysis). There was also a significant interaction between race and serum Na^+^ (*p* = 0.02). These data demonstrate an upward shift in the relation between serum Na^+^ and absolute systolic BP in African American adults. Categorical serum Na^+^ changes and corresponding changes in systolic BP throughout the HSI are presented in **Figure 2A**. There was a significant main effect for categorical serum Na^+^ changes (*p* = 0.008) and a significant main effect for group (*p* = 0.006) suggesting that as levels of serum sodium increased there was a greater increase in systolic BP, and that for a given categorical response in serum Na^+^, African American participants demonstrated a greater systolic BP response. Regarding categorical serum Na^+^ changes, 51% of African American participants had a “large” change in serum Na^+^ during HSI while 30% of Caucasian participants had a “large” change in serum Na^+^ during HSI (**Figure 2B**, *p* = 0.06).

**FIGURE 3:**
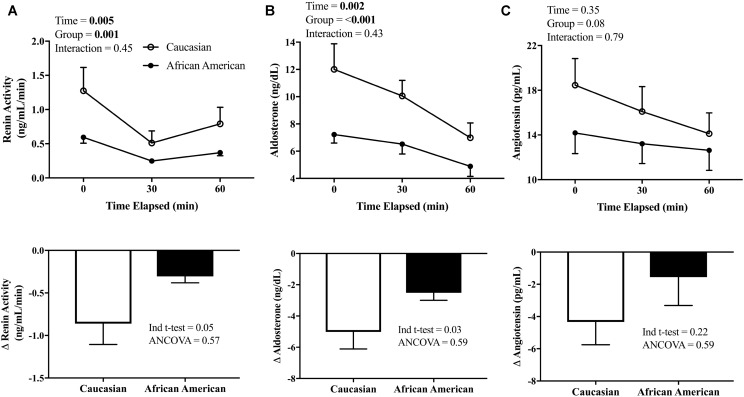
Renin-angiotensin-aldosterone system (RAAS) response to HSI. **(A)** PRA (African American *n* = 16, Caucasian *n* = 12) measured at baseline and throughout the HSI (top panel) and peak responses (bottom); **(B)** Aldo (African American *n* = 18, Caucasian *n* = 20) measured at baseline and throughout the HSI (top panel) and peak responses (bottom); and **(C)** Ang II (African American *n* = 16, Caucasian *n* = 12) measured at baseline and throughout the HSI (top panel) and peak responses (bottom). African American participants are represented in closed circles (•) and Caucasian participants in open circles (∘). Statistically significant main or interaction effect where *p* < 0.05. Data presented as mean ± SEM.

The RAAS hormones were corrected for the expansion in plasma volume, and are presented in **Figure 3**. PRA and Aldo decreased throughout the HSI in both African American and Caucasian participants (see **Figures 3A,B**, top panel; *p* < 0.01 main effect for time for both PRA and Aldo) and there was a significant main effect for race (*p* ≤ 0.001 for both). The peak reduction in PRA (*p* = 0.05) and Aldo (*p* = 0.03) from baseline to the end of the HSI (see **Figures 3A,B**, bottom panel) was greater in Caucasian participants when compared using an independent sample *t*-test. However, African American participants demonstrated lower baseline values of Aldo (*p* = 0.03) and there was a trend for lower PRA in African American participants compared to Caucasian participants (*p* = 0.07). Therefore, when the lower baseline PRA and Aldo for African American participants was factored in as a covariable, these differences were no longer statistically significant for either PRA (*p* = 0.56) or Aldo (*p* = 0.58). Regarding Ang II, there was not a significant main effect for time or race (**Figure 3C**, top panel). Further, the peak change in Ang II was not different between African American and Caucasian participants when compared using a *t*-test or ANCOVA (**Figure 3C**, bottom panel).

Norepinephrine was not increased over the HSI (231.7 ± 20.8 – 295.0 ± 34.6 pg/mL for African American adults and 218.0 ± 22.1 – 251.1 ± 24.1 pg/mL for Caucasian adults; main effect for time, *p* = 0.114) and there were no racial differences in NE concentrations at baseline or throughout the HSI (main effect for group; *p* = 0.185). The peak change in NE was also not different between African American and Caucasian participants when compared using a *t*-test or ANCOVA.

## Discussion

The purpose of this study was to determine whether serum Na^+^, BP, and hormonal responses to an acute Na^+^ and volume load via HSI differ between young normotensive African American and Caucasian adults. The primary novel findings were that there was a significant interaction between race and absolute serum Na^+^ on systolic BP. For a given change in serum Na^+^ with the HSI, African American individuals demonstrated a greater increase in systolic BP. Further, African American individuals were more likely to demonstrate large increases in serum Na^+^ and displayed a greater peak change in serum Na^+^. These findings are of importance in light of (a) racial differences in the prevalence of salt-sensitive hypertension (Williams et al., 2014; Fryar et al., 2017), and (b) the recent emphasis on plasma Na^+^ concentration and BP in the literature (He et al., 2005; He and Macgregor, 2012; Whelton et al., 2012). Additionally, several studies indicate that a single high Na^+^ meal can similarly increase plasma Na^+^ (Dickinson et al., 2011, 2014; Suckling et al., 2012; Blanch et al., 2015) and result in post-prandial reductions in endothelial functional (Dickinson et al., 2011, 2014; Blanch et al., 2015) and increases in BP (Suckling et al., 2012). It has also been shown that young healthy African American females retain more Na^+^ than young Caucasian females when placed on a high salt diet (Palacios et al., 2004). Thus, one may speculate that African American individuals could potentially be at greater risk for these negative post-prandial effects of increased plasma Na^+^, and over time, repeated exposure may contribute to the higher prevalence of salt-induced hypertension.

Consistent with the study hypothesis and previous literature (Luft et al., 1991), PRA and Aldo declined in response to the HSI; the decrease in PRA and Aldo was blunted in African American participants compared to Caucasian participants, indicating a less responsive RAAS in the face of an acute Na^+^ and volume load. However, this difference was no longer significant when we included the lower baseline PRA and Aldo values as covariables. Thus, it appears that not only the response of the RAAS is important, but also the baseline value, as there may be a floor effect due to this lower baseline. In other words, the lower baseline PRA and Aldo in African American individuals contribute to less of an absolute change in these hormones in response to Na^+^ challenges. It should be noted that previous studies reporting a greater reduction in PRA and Aldo in Caucasian cohorts compared to African American cohorts did not appear to use baseline values as covariables (Luft et al., 1991). This is an important consideration given that numerous studies have reported lower basal PRA and Aldo in African Americans individuals (Levy et al., 1977; Grim et al., 1979; Luft et al., 1979; Sever et al., 1979; He et al., 1998, 1999), including a recent publication from the Multi-Ethnic Study of Atherosclerosis (Rifkin et al., 2014). Further, another recent study demonstrated greater reductions in PRA and Aldo with aging in African American individuals (Tu et al., 2018). Taken together with our data, this is physiologically and clinically relevant given the increased prevalence of hypertension, salt sensitive hypertension, and cardiovascular disease in African American adults as they age.

The cause of lower circulating levels of the RAAS in African American populations remains unclear. Interestingly, potassium supplementation has been shown to increase PRA in African American individuals, indicating some of the difference in RAAS hormones could be influenced by dietary intake (Pratt et al., 1997). Thus, it would appear basal RAAS levels have important physiological consequences for buffering acute high Na^+^ challenges (Luft et al., 1991) and potentially chronic high Na^+^ diets (Luft et al., 1979). In addition to plasma RAAS concentrations, RAAS signaling at the tissue level has also been shown to play an important role in vascular health and disease. For example, increased intra-renal RAAS activity has been shown to contribute to an exaggerated reduction in renal plasma flow in healthy African American individuals compared to healthy Caucasian individuals during a high salt diet (Price et al., 2002).

One potential cause for lower RAAS in African American populations could be greater Na^+^ retention and expanded extracellular fluid volume (resulting in similar serum Na^+^ compared to other populations). Indeed, a previous study has shown that African American individuals demonstrate greater total body water (Chumlea et al., 2001). Our participants were given the same instructions for Na^+^ and water intake for the day before the experimental visit. Baseline serum sodium values were similar between groups, and increased during the HSI (see **Table 2**). For any given level of serum sodium, BP was greater in African American adults. Importantly, there was a significant main effect for categorical serum Na^+^ changes and a greater peak Na^+^ change with the HSI in African American participants, demonstrating that African American adults had higher serum Na^+^. Taken together, these findings suggest the African American participants had an impaired short-term ability to clear excess Na^+^. While we did not assess urinary Na^+^ excretion following the HSI, our findings are supported by a previous publication that reported African American individuals had reduced Na^+^ excretion in the first 12 h following an HSI compared to Caucasian individuals (Luft et al., 1991). Another study using incremental doses of salt for 3-day controlled diets found that African American individuals had reduced natriuresis compared to Caucasian individuals as dietary Na^+^ loads increased (Luft et al., 1979). Further, in recent years it has become appreciated that non-osmotic Na^+^ deposition in tissues (e.g., skin, muscle) also contributes to whole body Na^+^ balance and that sex and age influence tissue Na^+^ (Kopp et al., 2013; Wang et al., 2017). To the best of our knowledge, the role of race in tissue Na^+^ deposition remains unstudied, but may have important consequences in Na^+^ regulation.

The sympathetic nervous system may also play an important role in the BP responses we observed in the current study. Although dietary Na^+^ increases sensitivity to sympathoexcitatory stimuli in rodents (Adams et al., 2008; Stocker et al., 2010; Simmonds et al., 2014), the potential racial differences in humans remain unexplored. While we did not directly measure sympathetic nerve activity in this study, the HSI did not elicit a large increase in NE, an indirect measure of sympathetic outflow (Wallin et al., 1981). There were no differences in NE observed between the African American and Caucasian participants at baseline or throughout the HSI. However, recent data suggest that African American individuals demonstrate greater sympathetic transduction (Vranish et al., 2018). As noted in another recent study investigating racial differences in neural control of BP, habitual dietary patterns and stress (i.e., racial differences in allostatic load) could potentially influence racial differences in sympathetic BP regulation (Fonkoue et al., 2018). Therefore, it could be that for a given increase in SNA, the African American participants in this study demonstrated a greater increase in BP. Unfortunately, we did not directly measure sympathetic outflow or blood flow to assess transduction. Therefore, this remains speculative but is supported by our previous findings that HSI increases sympathetic outflow (Farquhar et al., 2006; Wenner et al., 2007) and the recent findings of heightened transduction in African American adults (Vranish et al., 2018).

We recognize our study is not without limitations. Although we instructed participants to refrain from salty foods prior to the experimental visit, sodium intake was not strictly controlled (i.e., controlled feeding study). However, our focus was on the response to the acute hypertonic saline load. Additionally, all participants were given the same instructions as to Na^+^ and water intake for the day before and morning of the experimental visit. Second, we did not assess arginine vasopressin during the HSI, which has previously been shown to increase in response to HSI (Thibonnier et al., 1999; Calzone et al., 2001; Stachenfeld and Keefe, 2002). Therefore, potential racial differences in arginine vasopressin could have contributed to our findings. Third, while the difference in sex distribution between the African American and Caucasian cohorts was not statistically significant, we recognize it could be meaningful. However, one might speculate the proportion of females in the African American cohort would lead to *less* BP responsiveness, as we have previously shown females are less susceptible to endothelial dysfunction following high dietary salt (Lennon-Edwards et al., 2014) and females demonstrate less salt sensitivity compared to male counterparts (Bursztyn and Ben-Dov, 2013; Elijovich et al., 2016). Nonetheless, we observed greater BP responses for a given increase in serum sodium in the African American cohort. Finally, we also did not use an isovolumic isotonic control in this study. Importantly, we have previously shown that extracellular volume expansion alone does not elicit the same cardiovascular alterations that HSI does (Farquhar et al., 2005, 2006). Nonetheless, we report potentially important racial differences in BP regulation in response to increases in serum Na^+^. Despite this, we cannot dismiss the possibility that racial differences exist in response to extracellular volume expansion alone.

In conclusion, acute increases in serum sodium via hypertonic saline resulted in greater BP responsiveness in African American participants for a given level of serum Na^+^. The ability to buffer the challenge may have been blunted in African American individuals due to a lower resting circulating levels of the RAAS. This resulted in less of a reduction in PRA and Aldo to the HSI. African American and Caucasian participants demonstrated a similar increase in NE; however recent evidence suggests there could be heightened sympathetic transduction in the African American participants (Vranish et al., 2018). These findings are important as they suggest that African American individuals may not be able to buffer acute Na^+^ challenges as Caucasian individuals. These findings have important clinical implications: acute elevations in serum Na^+^ (i.e., post-prandial) have a negative impact on the health of the vasculature (Dickinson et al., 2011, 2014; Suckling et al., 2012; Blanch et al., 2015), and our data demonstrate important racial differences in BP responsiveness. Thus, it is likely that repeated increases in BP responsiveness over time may contribute to the racial disparity in salt-sensitive hypertension.

## Author Contributions

MW, EP, and WF performed the experiments. EP and AR analyzed the data and prepared the figures. WF contributed to the conception and design of the research. MW, EP, and AR drafted the manuscript. MW, EP, AR, WR, and WF interpreted the results of the experiments, edited and revised the manuscript, and approved the final version of the manuscript.

## Conflict of Interest Statement

The authors declare that the research was conducted in the absence of any commercial or financial relationships that could be construed as a potential conflict of interest.

## Abbreviations

Aldo: aldosterone
Ang II: angiotensin II
BP: blood pressure
HSI: hypertonic saline infusion
NE: norepinephrine
PRA: plasma renin activity
RAAS: renin-angiotensin-aldosterone system
